# Ultrasound in Infertility Setting: Optimal Strategy to Evaluate the Assessment of Tubal Patency

**DOI:** 10.1155/2017/3205895

**Published:** 2017-12-11

**Authors:** Luca Mandia, Carlo Personeni, Patrizio Antonazzo, Salvatore Alessio Angileri, Antonio Pinto, Valeria Savasi

**Affiliations:** ^1^Reproductive Unit, Clinic of Obstetrics and Gynecology, Department of Biomedical and Clinical Sciences, ASST Fatebenefratelli Sacco, Hospital “L. Sacco”, University of Milan, Milan, Italy; ^2^Diagnostic and Interventional Radiology Unit, Department of Health Sciences, University of Milan, Milan, Italy; ^3^Department of Radiology, Cardarelli Hospital, Naples, Italy

## Abstract

Tubal patency is a key element in women who are undergoing assisted reproductive techniques (ART), in order to attempt or exclude intrauterine insemination (IUI) cycles. Amongst the different procedures that can be used, without resorting to laparoscopy that remains the gold standard, hystero-salpingo-contrast sonography (HyCoSy) is an acceptable, time-efficient, and well tolerated option; it can be performed with administration of saline and air simultaneously or alternately (air/saline-HyCoSy), or with some other contrast agents, like SonoVue (sulfur hexafluoride microbubbles). In this paper, we describe two different studies: in the first one, our aim is to compare the efficiency of air/saline-HyCoSy with HyCoSy performed with contrast media (SonoVue), considering hysterosalpingography (HSG) and laparoscopy (LPS) as reference tests; in the second one, we estimate the pregnancy rate of a cohort of infertile women selected to undergo IUI cycles after tubal bilateral patency demonstration with air/saline-HyCoSy, to understand if this technique can be used as an efficient screening procedure in a Reproductive Unit.

## 1. Introduction

Assessment of tubal patency is a first-step examination in the evaluation of the infertile women. Laparoscopy with chromopertubation is considered the gold standard test for tubal assessment, adding hysteroscopy to the procedure for concomitant evaluation of the intrauterine cavity. Laparoscopy thus can lead to a final assessment of pelvic factors; however, even this direct visualization technique never reaches a perfect correlation with fertility outcome and it exposes patients to potential surgical complications [1, 2]. Besides, noninvasive techniques can be used to assess tubal patency. Hysterosalpingography (HSG) is still the first-line diagnostic examination in some fertility centers, but it also shows limits and potential risks: on one side, it enables poor or no evaluation of the uterus and the ovaries; on the other side, it exposes to ionizing radiation and potentially allergenic contrast media. The introduction of hystero-salpingo-contrast sonography (HyCoSy) provides an increasingly common alternative, proven to be an acceptable, time-efficient, and well tolerated option to HSG with comparable accuracy in the assessment of the uterine cavity and tubal patency [3, 4]. HyCoSy can be performed with administration of saline and air simultaneously or alternately (air/saline-HyCoSy), or with some other contrast agents [5]. Some investigators [6, 7] have demonstrated the limitations of conventional two-dimensional HyCoSy (2D-HyCoSy), which are mainly due to the fact that the fallopian tubes are rarely visualized completely because of their anatomical tortuosity. Furthermore, spill of the positive medium from the distal ostium could be difficult to distinguish from the surrounding bowels. To overcome these limits, three-dimensional imaging with HyCoSy has been proposed [8]. We performed two studies. In the first one the main purpose was to evaluate the efficacy of contrast-tuned imaging technology (CnTI-Sono Vue-HyCoSy) compared to air/saline-HyCoSy, considering both laparoscopy and hysterosalpingography as diagnostic reference standards. In the second one the aim was to compare the pregnancy rate in infertile women undergoing air/saline-HyCoSy and intrauterine insemination to the pregnancy rate in fertile population.

## 2. Materials and Methods

We performed two different studies in order to evaluate the tubal patency in women with infertility problems.

### 2.1. Study One

Forty-two patients undergoing investigation for subfertility underwent HyCoSy during the proliferative phase of the menstrual cycle. Transvaginal ultrasound was performed using a Technos MPX (ESAOTE SpA, Genova, Italy) ultrasound machine and a high-resolution (5.0–9.0 MHz) endovaginal probe. A basic scanning was performed to evaluate any abnormal findings of the uterus and ovaries. Informed consent was obtained from all patients according to local Institutional Review Board procedures.

**Figure 1:**
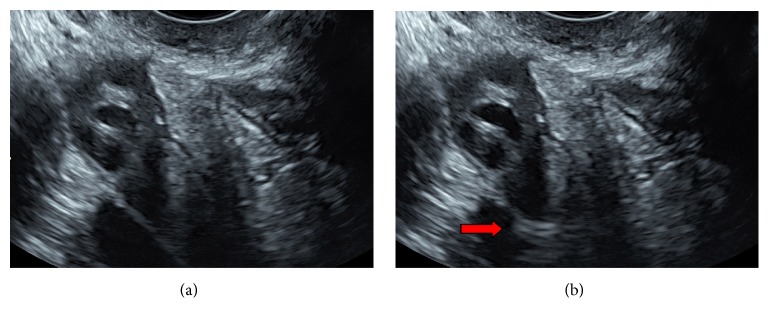
*Assessment of tubal patency with air/saline hysterosalpingography*. (a) shows the image of anexa with US contrast. (b) Red arrows show the hyperechoic echo of air/saline contrast media.

After accurate cleaning of the cervix and vagina with an iodinated solution, an 8-Fr catheter (pediatric Nelathon; Teleflex Medical S.r.l., Varedo, Italy) was introduced transcervically. After successful catheter placement, the two diagnostic procedures were performed sequentially. We started with a HyCoSy using saline with air as contrast medium (air/saline-HyCoSy) and ultrasound performed on the 2D setting and B-mode. Five to ten millilitres of sterile saline were first injected into the uterine cavity to determine whether there were any intracavitary abnormalities; subsequently, 30 mL of air was gently injected to induce the bubbling effect and visualize the positive signal in the tubal lumen (Figure 1). With the same catheter, we performed the second HyCoSy using SonoVue as contrast media (sulfur hexafluoride microbubbles; Bracco Imaging S.p.a., Milan, Italy) and scanning using the CnTI-SonoVue-HyCoSy. The CnTI-technology works at very low acoustic pressure (Mechanical index < 0.1). At such a low acoustic pressure, tissues do not exhibit any harmonic response, while microbubbles give a signal that is picked up by the transducer [9]. Second-generation contrast media use very stable microbubbles, with minimal bubble destruction [10]. We diluted 4.8 mL of SonoVue with 20 mL of saline and used 5–10 mL of the final solution for each patient.

No antispasmodic drugs were used. If the patients had a recent (30 days) negative cervical swab for chlamydia and mycoplasma and negative vaginal swab, no antibiotics were administered. Otherwise, we opted for antibiotic prophylaxis with doxycycline (doxycycline, 200 mg) one-half hour before the procedure or azithromycin (Azithromycin, 500 mg) starting 2 days before the procedure and finishing on the day of the procedure.

In this series of patients, the final diagnosis was obtained within 2 months from the HyCoSy, either by conventional HSG or by LPS in selected cases. The diagnosis of tubal patency for saline and B-mode ultrasound was obtained when the following criteria were met: (1) visualization of the flow of contrast media in the tubes without dilatation of the lumen; (2) appearance of contrast media in the peritoneal cavity near the ovaries (hyperechoic image).

The diagnosis of tubal patency for CnTI-SonoVue-Hy- CoSy was obtained with the direct display of the contrast medium first in the uterine cavity and immediately thereafter in the tubes. Evocation of pain during the distension of the uterine cavity without visualization of flow in the tubes was an additional criterion of organ damage.

### 2.2. Study Two

Two hundred infertile patients were analysed with air/saline-HyCoSy with the same procedure described in study one. Data were recorded and analysed. In the presence of a bilateral patency, patients were sent to the Reproductive Unit for intrauterine insemination. In the presence of at least one occluded tube, patients were admitted to an IVF cycle. Couples admitted to IUI underwent ovulation induction using gonadotropins. A low dose (75 IU) (Gonal F®, Ares-Serono, UK or Puregon®, Organon, France) of recombinant FSH was given from day 3 to the day of ovulation induction (HCG 5000 IU) based on follicle ultrasound monitoring from day 8 to a dominant follicle of 18 mm mean diameter. Insemination was performed 36 h after HCG administration. For the IUI, we use only fresh sperm; inclusion criteria for this study were as follows: no relevant systematic disease, body mass index ≤ 30 kg/m^2^, normal karyotype, and normal follicular stimulating hormone (FSH), luteinizing hormone (LH), prolactin (PRL), and thyroid-stimulating hormone (TSH). In couples admitted to IVF, FSH injections for multifollicular ovarian stimulation were started at a dose of 150–300 IU every day. Final oocyte maturation was induced using 10,000 IU of human chorionic gonadotropin when at least three 18 mm follicles were seen on ultrasound scan. Ultrasound guided oocyte retrieval was done 34–38 h after human chorionic gonadotropin administration. Progesterone supplementation was used for luteal phase support and continued for up to 8 weeks of gestation if pregnancy had occurred. Embryo transfer was performed under transabdominal ultrasound guidance 2, 3, or 5 days after oocyte retrieval depending on the number and quality of the embryos available and the patient's will.

### 2.3. Statistical Analysis

We considered the presence of an occluded tube as testing positive and the presence of a patent tube as negative. Inconclusive examinations were recorded. To assess the diagnostic accuracy, these examinations were grouped together with the occluded group. Parametric descriptive statistics were used for demographic data. The diagnostic accuracy of the two techniques was calculated, and Cohen's kappa value was used to compare the results of different examinations. The diagnostic accuracy of air/saline-HyCoSy and CnTI-SonoVue- HyCoSy obtained in the second half of this series was compared with the results obtained in the first half to evaluate the possible impact of a learning phase.

## 3. Results

### 3.1. Study One

The mean age of patients enrolled for this study was 35 years, with an SD of 4.5 years. Of the 42 patients enrolled, 26 patients underwent HSG and 16 patients underwent LPS to assess tubal patency and to guide clinical management.

In Table 1, we report the results of the air/saline-HyCoSy compared with these examinations. Air/saline-HyCoSy had a sensitivity, specificity, and positive and negative predictive value of 91%, 71%, 55%, and 95%, respectively. Seven patients had bilateral occlusion at HyCoSy, two of them had bilateral patency at LPS, and the other five cases confirmed bilateral occlusion. In Table 2, we report the results of the CnTI-SonoVue-HyCoSy compared with HSG/LPS. CnTI-SonoVue-HyCoSy had a sensitivity, specificity, and positive and negative predictive value of 87%, 84%, 69%, and 94%, respectively. Four patients had bilateral occlusion at CnTI-SonoVue; only one was not confirmed and showed a bilateral patency at LPS. The diagnostic accuracy of air/saline-HyCoSy and CnTI-SonoVue-HyCoSy compared with HSG/LPS were 77% and 85%, with a Cohen's kappa of 0.52 and 0.66, respectively.

Finally, we evaluated the kappa value and the diagnostic accuracy for air/saline-HyCoSy and CnTI-SonoVue-HyCoSy calculated in the first half (*n* = 41) and in the second half (*n* = 40) of examinations. The diagnostic accuracy of air/saline-HyCoSy in the two subsequent series improved from 70% to 80%, while that for CnTI-SonoVue-HyCoSy did not increase and was 85% in both series. The kappa value of air/saline-HyCoSy improved from 0.42 to 0.57, while for CnTI-SonoVue-HyCoSy it did not significantly change and was 0.68 in the first half and 0.64 in the second half of examinations. No pelvic infection occurred in any patient after the two-step HyCoSy and HSG or LPS.

### 3.2. Study Two

We enrolled two hundred infertile patients who underwent IUI and met all inclusion criteria described before. In this group mean women age was 36 ± 4.9 years and mean BMI was 23 ± 3.0 (range 16.8–30) Kg/m^2^. Mild asthenoteratozoospermia and male infection for HIV or HCV infection were infertility causes for the majority of couples (*n* = 140) and unexplained infertility was the second cause (*n* = 60). Performing air/saline-HyCoSy we found that 154 patients (77%) showed bilateral patency; in 18 cases (9%), no contrast medium was seen, and we considered them bilateral tubal occlusion; in 28 cases (14%), only monolateral passage was seen. 336 (84%) tubes were patent, 61 (15,3%) were occluded, and 3 (0,7%) were absent for previous surgery. 154 couples underwent superovulation and IUI. The mean number of treatments per couple was 4.13. 70% of pregnancies were conceived in the first three cycles. Six (3%) pregnancies were multiple. The pregnancy rate for couple up to 6 cycles was 78% and for IUI cycles was 20%. No cases of ectopic pregnancy were registered.

## 4. Discussion

During the evaluation of an infertility couple one of the crucial point is to understand if the tubes are patent or not in order to decide which type of procedure we have to perform. Laparoscopy is considered the gold standard but, obviously, this cannot be considered as a screening test. We tried to determine which test for tubal patency is more tolerable for patients, less expensive, and with a good sensibility. This study analyses the possible advantages of different techniques that combine second-generation contrast media with a dedicated sonographic technology and in the second study air/saline-HyCoSy as an efficient test to screen patients that desire a child through a Reproductive Program is analysed. Our results show that the kappa value of CnTI-SonoVue HyCoSy reaches an efficient agreement (kappa 0.66) with the final diagnosis, whereas the traditional air/saline-Hy-CoSy yielded a kappa value of 0.52, corresponding to moderate agreement with the reference standard. This latter value obtained by air/saline-HyCoSy confirms most of the literature reporting these techniques [11–16]. The very high normal predictive value (95%) of air/saline-HyCoSy may suggest that this procedure can be performed as a screening examination to avoid the use of more expensive contrast media. In our results, inconclusive examinations were grouped together with the “occluded” group. The positive predictive value, excluding these inconclusive cases, improved from 55% to 72% for air/saline-HyCoSy and from 69% to 80% for CnTI-SonoVue-HyCoSy, with a Cohen's kappa of 0.70 and 0.76, respectively. Considering the two techniques comparable for efficiency, in the second part of our study we performed only air/saline-HyCoSy. Our results show that pregnancy rate in a group of subfertile patients with established patent tubes is comparable to the pregnancy rate in the fertile population similar for female age. These results, although indirectly, suggest that air/saline-HyCoSy has a good specificity and we can consider air/saline-HyCoSy an ideal one-step procedure to assess tubal patency, also considering the minor cost, due to the use of saline only and no need of dedicated 3D software.

In study one, we perform the two sequential techniques during the same examination with the same catheter, avoiding any additional discomfort to the patient. These second-generation contrast media that are used with the tuned imaging technology can be proposed in cases of positive diagnoses of tubal occlusion or in inconclusive results. By means of CnTI-SonoVue-HyCoSy, we obtained a sensitivity and specificity of 87% and 84%, respectively. Our results are in agreement with other investigators who reported their findings on smaller series [16, 17], showing that positive contrast agents are more efficient than saline solution. In addition to this, our results show that the use of second-generation contrast media does not require a learning period as observed in our series for air/saline-HyCoSy [17]. We can explain this by the long persistence of a positive contrast agent versus the short imaging effect of saline boosted by air injection, since bidimensional ultrasound requires the evaluation of different sections to spot the whole course of fallopian tubes. Contrast agents persistently “paint” the tubes and make it easier and more reliable for the sonographer to come to a final diagnosis.

However, it is important to distinguish the clinical meaning of “tubal factor” and “tubal patency” since patency does not imply a normal function of the fallopian tubes. Tubal peristalsis and integrity of the ciliate epithelium are mandatory to permit the oocyte fertilization and a normal intrauterine implantation of the embryo. This fine synchronization represents one of the key factors for successful pregnancy, and its absence would explain a part of idiopathic infertility. From this perspective, macroscopic imaging of tubal factor, whatever its accuracy in confirming tubal patency, does not represent a final assessment of the tubal factor. LPS provides an accurate assessment of the pelvis and the characteristics of the tubes and its ligaments, but tubal patency is eventually proved by chromopertubation obtained after major stress applied to the uterine cavity by a hysteron manipulator. Tubal spasm and selected sided spill, which imbalances tubal resistance, can occur at the LPS assessment of tubal patency as well as during more gentle procedures such as HSG and HyCoSy.

## 5. Conclusions

CnTI-SonoVue-HyCoSy is more accurate than air/saline-HyCoSy for the assessment of fallopian tubes. The very high normal predictive value of air/saline-HyCoSy suggests that this procedure could be performed as a screening examination, whereas CnTI-SonoVue-HyCoSy could be used as a second-step technique. In a subfertile population, air/saline-HyCoSy proves to be an efficient screening method to attempt tubal patency to select patients who can undergo intrauterine insemination as first step of a medical assisted procreation program.

## Figures and Tables

**Table 1:** Air/saline-HyCoSy versus HSG/LPS (81 tubes, 42 patients).

Air/saline-HyCoSy	HSG/LPS		
	Patent	Occluded	Total
Patent	41	2	43
Occluded	17	21	38

Total	58	23	81

**Table 2:** CnTI-SonoVue-HyCoSy versus HSG/LPS (81 tubes, 42 patients).

CnTI-SonoVue-HyCoSy	HSG/LPS		
	Patent	Occluded	Total
Patent	49	3	52
Occluded	9	20	29

Total	58	23	81
